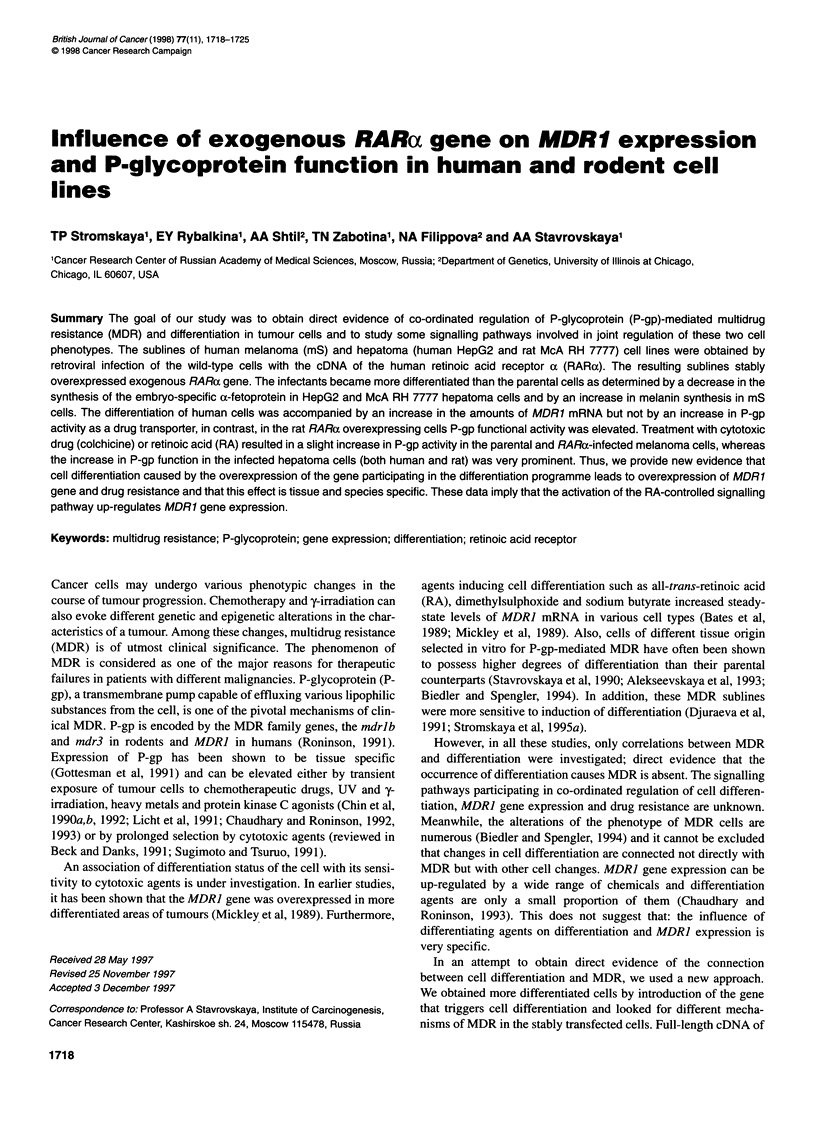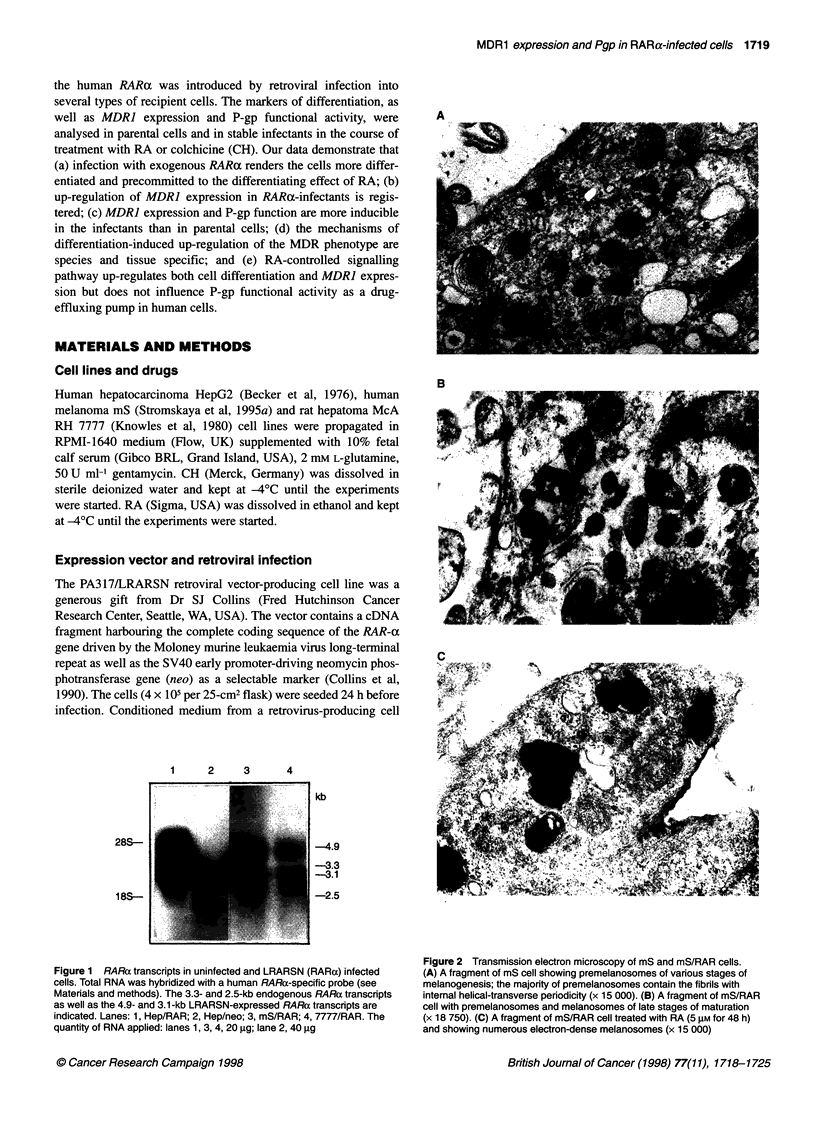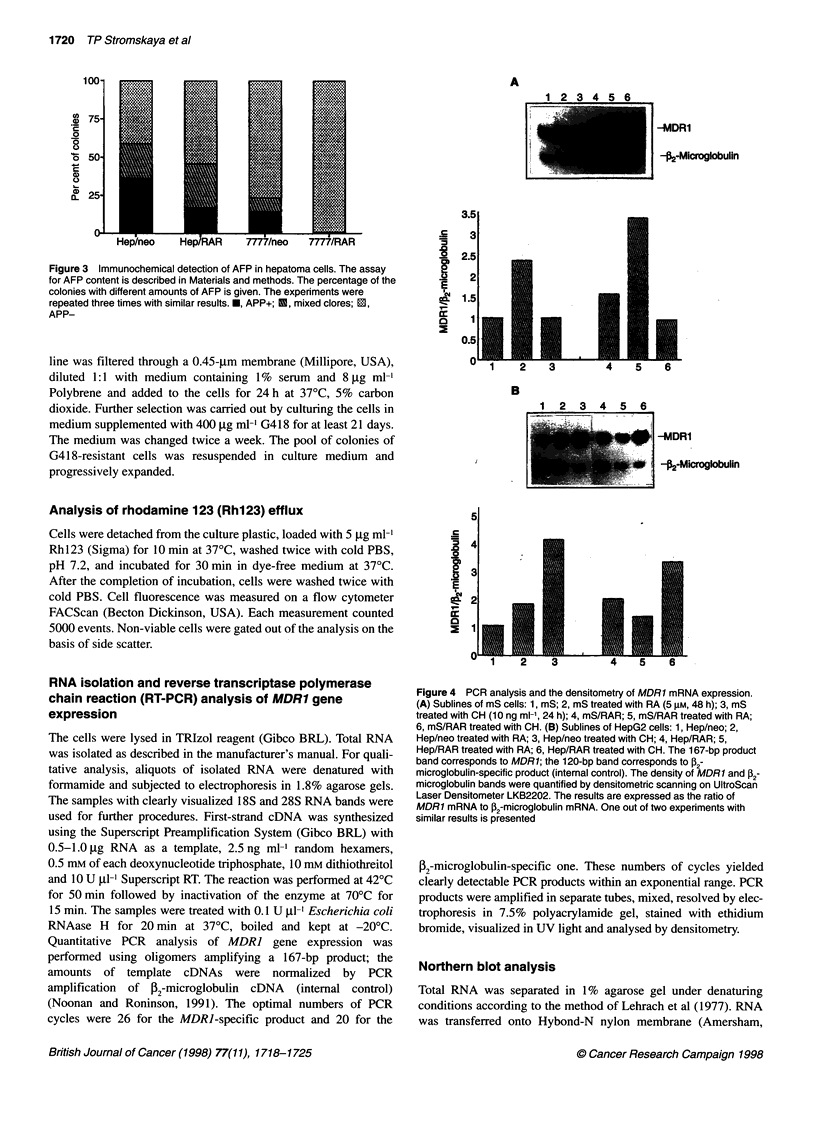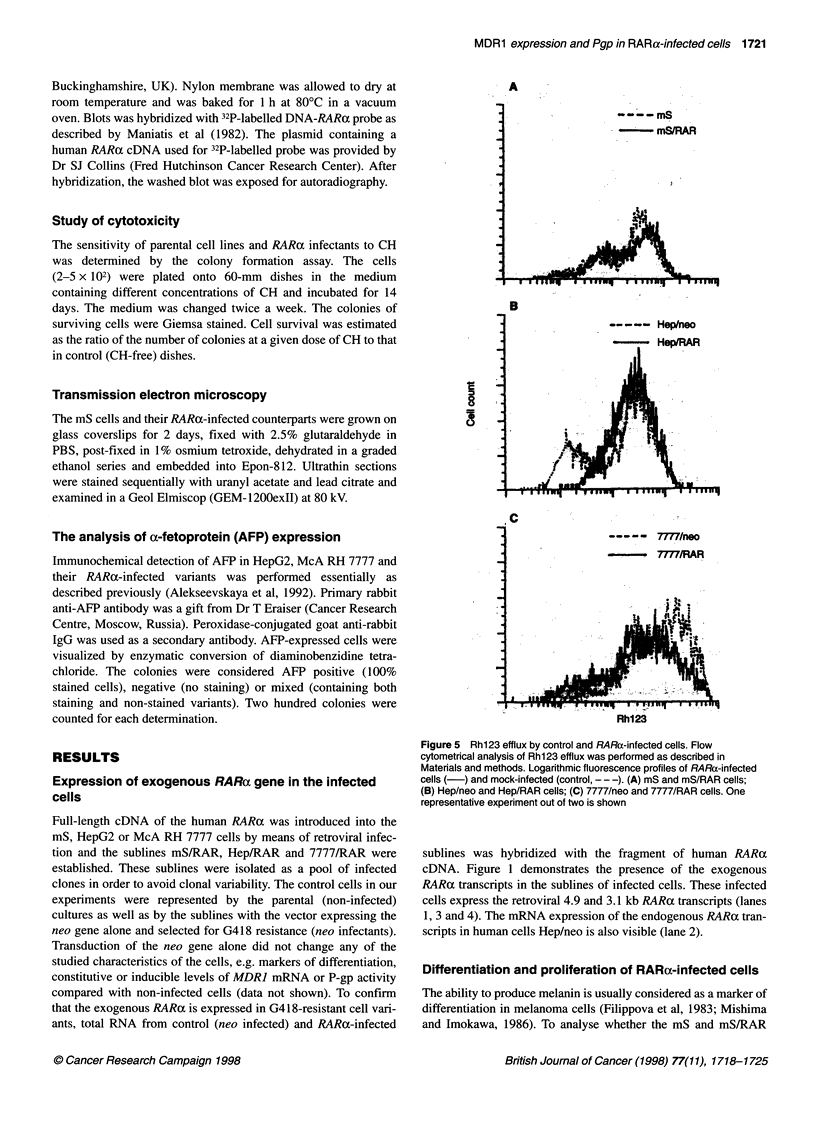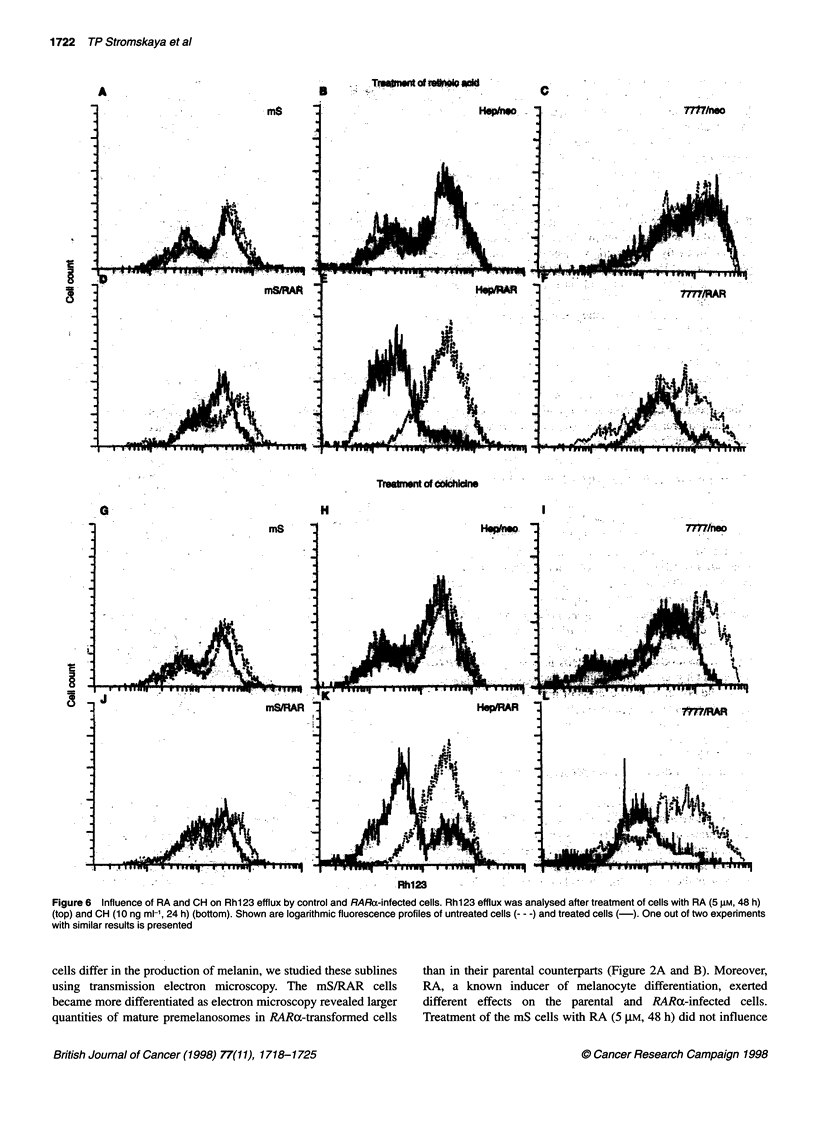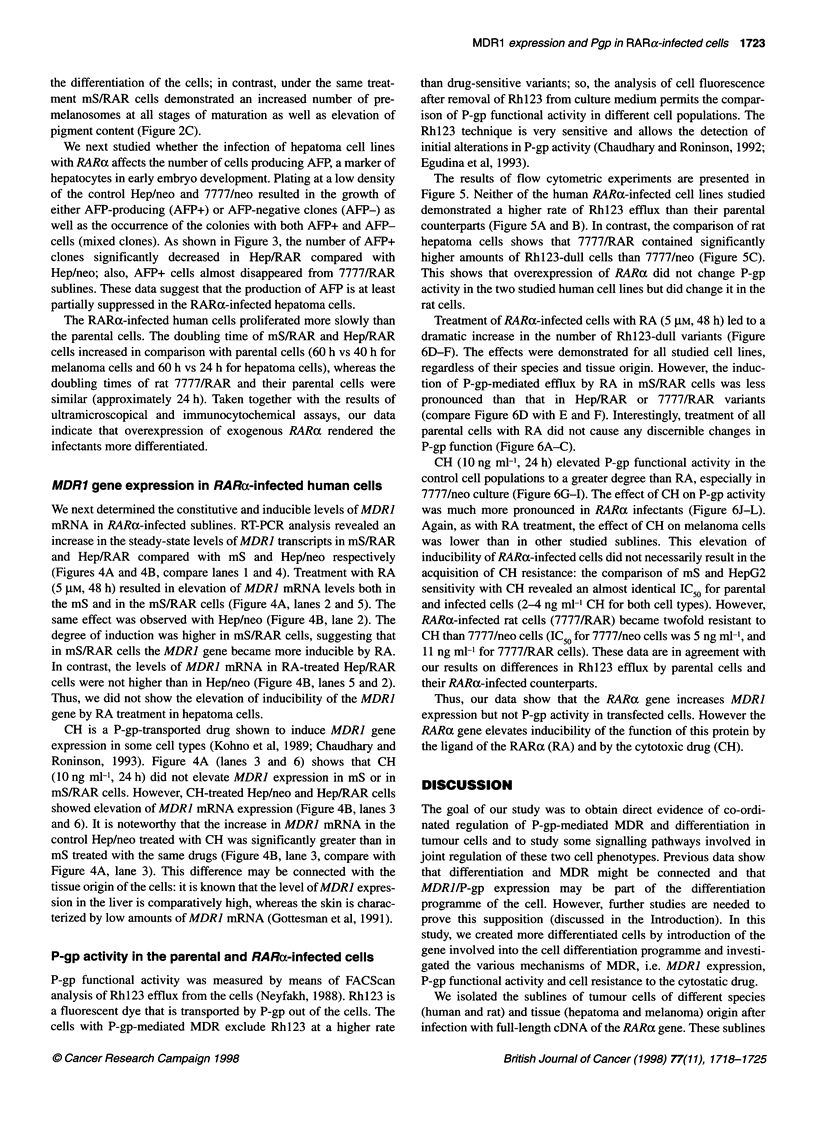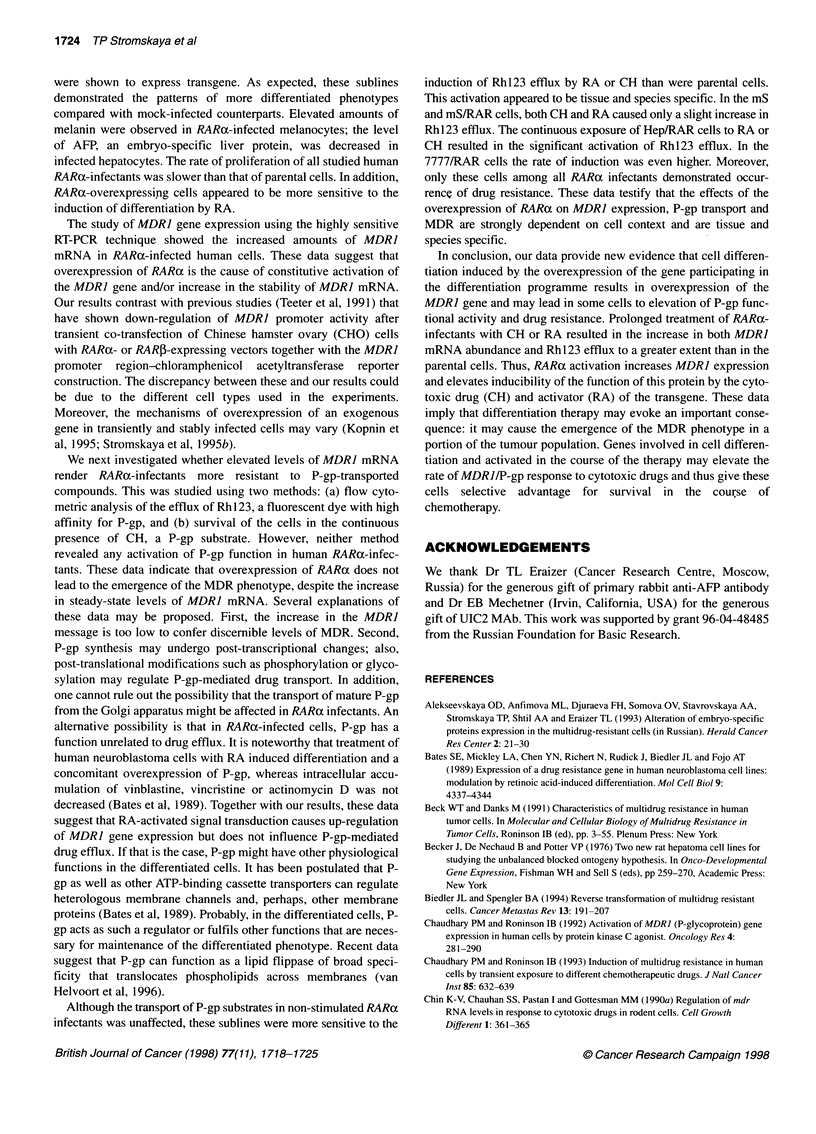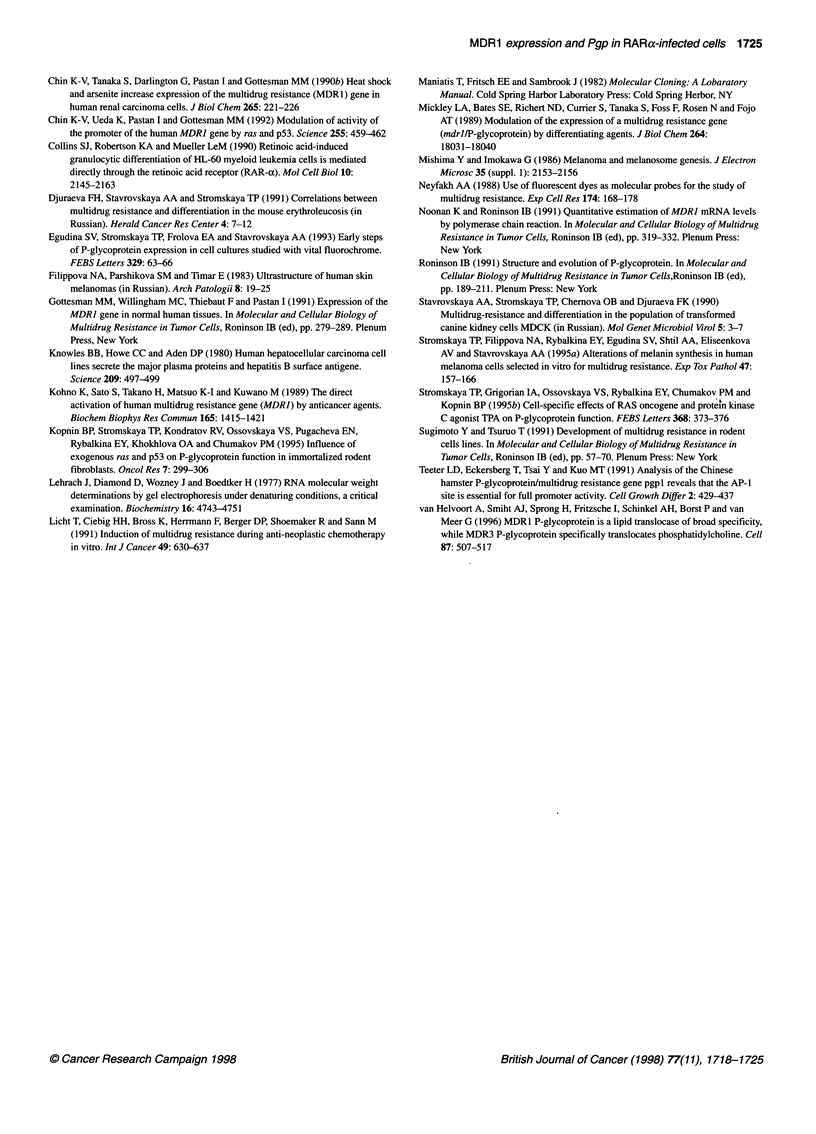# Influence of exogenous RAR alpha gene on MDR1 expression and P-glycoprotein function in human and rodent cell lines.

**DOI:** 10.1038/bjc.1998.288

**Published:** 1998-06

**Authors:** T. P. Stromskaya, E. Y. Rybalkina, A. A. Shtil, T. N. Zabotina, N. A. Filippova, A. A. Stavrovskaya

**Affiliations:** Cancer Research Center of Russian Academy of Medical Sciences, Moscow.

## Abstract

**Images:**


British Journal of Cancer (1998) 77(11), 1718–1725
© 1998 Cancer Research Campaign

### Influence of exogenous *RAR*α gene on *MDR1* expression and P-glycoprotein function in human and rodent cell lines

**TP Stromskaya[1], EY Rybalkina[1], AA Shtil[2], TN Zabotina[1], NA Filippova[2] and AA Stavrovskaya[1]**

[1]Cancer Research Center of Russian Academy of Medical Sciences, Moscow, Russia; [2]Department of Genetics, University of Illinois at Chicago, Chicago, IL 60607, USA

**Summary** The goal of our study was to obtain direct evidence of co-ordinated regulation of P-glycoprotein (P-gp)-mediated multidrug resistance (MDR) and differentiation in tumour cells and to study some signalling pathways involved in joint regulation of these two cell phenotypes. The sublines of human melanoma (mS) and hepatoma (human HepG2 and rat McA RH 7777) cell lines were obtained by retroviral infection of the wild-type cells with the cDNA of the human retinoic acid receptor α (RARα). The resulting sublines stably overexpressed exogenous *RAR*α gene. The infectants became more differentiated than the parental cells as determined by a decrease in the synthesis of the embryo-specific α-fetoprotein in HepG2 and McA RH 7777 hepatoma cells and by an increase in melanin synthesis in mS cells. The differentiation of human cells was accompanied by an increase in the amounts of *MDR1* mRNA but not by an increase in P-gp activity as a drug transporter, in contrast, in the rat *RAR*α overexpressing cells P-gp functional activity was elevated. Treatment with cytotoxic drug (colchicine) or retinoic acid (RA) resulted in a slight increase in P-gp activity in the parental and *RAR*α-infected melanoma cells, whereas the increase in P-gp function in the infected hepatoma cells (both human and rat) was very prominent. Thus, we provide new evidence that cell differentiation caused by the overexpression of the gene participating in the differentiation programme leads to overexpression of *MDR1* gene and drug resistance and that this effect is tissue and species specific. These data imply that the activation of the RA-controlled signalling pathway up-regulates *MDR1* gene expression.

**Keywords:** multidrug resistance; P-glycoprotein; gene expression; differentiation; retinoic acid receptor

Cancer cells may undergo various phenotypic changes in the course of tumour progression. Chemotherapy and γ-irradiation can also evoke different genetic and epigenetic alterations in the characteristics of a tumour. Among these changes, multidrug resistance (MDR) is of utmost clinical significance. The phenomenon of MDR is considered as one of the major reasons for therapeutic failures in patients with different malignancies. P-glycoprotein (P-gp), a transmembrane pump capable of effluxing various lipophilic substances from the cell, is one of the pivotal mechanisms of clinical MDR. P-gp is encoded by the MDR family genes, the *mdr1b* and *mdr3* in rodents and *MDR1* in humans (Roninson, 1991). Expression of P-gp has been shown to be tissue specific (Gottesman et al, 1991) and can be elevated either by transient exposure of tumour cells to chemotherapeutic drugs, UV and γ-irradiation, heavy metals and protein kinase C agonists (Chin et al, 1990*a*,*b*, 1992; Licht et al, 1991; Chaudhary and Roninson, 1992, 1993) or by prolonged selection by cytotoxic agents (reviewed in Beck and Danks, 1991; Sugimoto and Tsuruo, 1991).

An association of differentiation status of the cell with its sensitivity to cytotoxic agents is under investigation. In earlier studies, it has been shown that the *MDR1* gene was overexpressed in more differentiated areas of tumours (Mickley et al, 1989). Furthermore, agents inducing cell differentiation such as all-*trans*-retinoic acid (RA), dimethylsulphoxide and sodium butyrate increased steady-state levels of *MDR1* mRNA in various cell types (Bates et al, 1989; Mickley et al, 1989). Also, cells of different tissue origin selected in vitro for P-gp-mediated MDR have often been shown to possess higher degrees of differentiation than their parental counterparts (Stavrovskaya et al, 1990; Alekseevskaya et al, 1993; Biedler and Spengler, 1994). In addition, these MDR sublines were more sensitive to induction of differentiation (Djuraeva et al, 1991; Stromskaya et al, 1995*a*).

However, in all these studies, only correlations between MDR and differentiation were investigated; direct evidence that the occurrence of differentiation causes MDR is absent. The signalling pathways participating in co-ordinated regulation of cell differentiation, *MDR1* gene expression and drug resistance are unknown. Meanwhile, the alterations of the phenotype of MDR cells are numerous (Biedler and Spengler, 1994) and it cannot be excluded that changes in cell differentiation are connected not directly with MDR but with other cell changes. *MDR1* gene expression can be up-regulated by a wide range of chemicals and differentiation agents are only a small proportion of them (Chaudhary and Roninson, 1993). This does not suggest that: the influence of differentiating agents on differentiation and *MDR1* expression is very specific.

In an attempt to obtain direct evidence of the connection between cell differentiation and MDR, we used a new approach. We obtained more differentiated cells by introduction of the gene that triggers cell differentiation and looked for different mechanisms of MDR in the stably transfected cells. Full-length cDNA of

*Received 28 May 1997*
*Revised 25 November 1997*
*Accepted 3 December 1997*

*Correspondence to:* Professor A Stavrovskaya, Institute of Carcinogenesis, Cancer Research Center, Kashirskoe sh. 24, Moscow 115478, Russia the human *RARα* was introduced by retroviral infection into several types of recipient cells. The markers of differentiation, as well as *MDR1* expression and P-gp functional activity, were analysed in parental cells and in stable infectants in the course of treatment with RA or colchicine (CH). Our data demonstrate that (a) infection with exogenous *RARα* renders the cells more differentiated and precommitted to the differentiating effect of RA; (b) up-regulation of *MDR1* expression in *RARα*-infectants is registered; (c) *MDR1* expression and P-gp function are more inducible in the infectants than in parental cells; (d) the mechanisms of differentiation-induced up-regulation of the MDR phenotype are species and tissue specific; and (e) RA-controlled signalling pathway up-regulates both cell differentiation and *MDR1* expression but does not influence P-gp functional activity as a drug-effluxing pump in human cells.

### MATERIALS AND METHODS

### Cell lines and drugs

Human hepatocarcinoma HepG2 (Becker et al, 1976), human melanoma mS (Stromskaya et al, 1995*a*) and rat hepatoma McA RH 7777 (Knowles et al, 1980) cell lines were propagated in RPMI-1640 medium (Flow, UK) supplemented with 10% fetal calf serum (Gibco BRL, Grand Island, USA), 2 mM L-glutamine, 50 U $ml^{-1}$ gentamycin. CH (Merck, Germany) was dissolved in sterile deionized water and kept at –4°C until the experiments were started. RA (Sigma, USA) was dissolved in ethanol and kept at –4°C until the experiments were started.

### Expression vector and retroviral infection

The PA317/LRARSN retroviral vector-producing cell line was a generous gift from Dr SJ Collins (Fred Hutchinson Cancer Research Center, Seattle, WA, USA). The vector contains a cDNA fragment harbouring the complete coding sequence of the *RAR-α* gene driven by the Moloney murine leukaemia virus long-terminal repeat as well as the SV40 early promoter-driving neomycin phosphotransferase gene (*neo*) as a selectable marker (Collins et al, 1990). The cells ($4 \times 10^5$ per 25-$cm^2$ flask) were seeded 24 h before infection. Conditioned medium from a retrovirus-producing cell

**Figure 1** *RARα* transcripts in uninfected and LRARSN (RARα) infected cells. Total RNA was hybridized with a human *RARα*-specific probe (see Materials and methods). The 3.3- and 2.5-kb endogenous *RARα* transcripts as well as the 4.9- and 3.1-kb LRARSN-expressed *RARα* transcripts are indicated. Lanes: 1, Hep/RAR; 2, Hep/neo; 3, mS/RAR; 4, 7777/RAR. The quantity of RNA applied: lanes 1, 3, 4, 20 μg; lane 2, 40 μg

**Figure 2** Transmission electron microscopy of mS and mS/RAR cells. (**A**) A fragment of mS cell showing premelanosomes of various stages of melanogenesis; the majority of premelanosomes contain the fibrils with internal helical-transverse periodicity (× 15 000). (**B**) A fragment of mS/RAR cell with premelanosomes and melanosomes of late stages of maturation (× 18 750). (**C**) A fragment of mS/RAR cell treated with RA (5 μM for 48 h) and showing numerous electron-dense melanosomes (× 15 000)

© Cancer Research Campaign 1998

**Figure 3** Immunochemical detection of AFP in hepatoma cells. The assay for AFP content is described in Materials and methods. The percentage of the colonies with different amounts of AFP is given. The experiments were repeated three times with similar results. ■, APP+; ▧, mixed clores; ▨, APP–

line was filtered through a 0.45-μm membrane (Millipore, USA), diluted 1:1 with medium containing 1% serum and 8 μg $ml^{-1}$ Polybrene and added to the cells for 24 h at 37°C, 5% carbon dioxide. Further selection was carried out by culturing the cells in medium supplemented with 400 μg $ml^{-1}$ G418 for at least 21 days. The medium was changed twice a week. The pool of colonies of G418-resistant cells was resuspended in culture medium and progressively expanded.

### Analysis of rhodamine 123 (Rh123) efflux

Cells were detached from the culture plastic, loaded with 5 μg $ml^{-1}$ Rh123 (Sigma) for 10 min at 37°C, washed twice with cold PBS, pH 7.2, and incubated for 30 min in dye-free medium at 37°C. After the completion of incubation, cells were washed twice with cold PBS. Cell fluorescence was measured on a flow cytometer FACScan (Becton Dickinson, USA). Each measurement counted 5000 events. Non-viable cells were gated out of the analysis on the basis of side scatter.

### RNA isolation and reverse transcriptase polymerase chain reaction (RT-PCR) analysis of *MDR1* gene expression

The cells were lysed in TRIzol reagent (Gibco BRL). Total RNA was isolated as described in the manufacturer's manual. For qualitative analysis, aliquots of isolated RNA were denatured with formamide and subjected to electrophoresis in 1.8% agarose gels. The samples with clearly visualized 18S and 28S RNA bands were used for further procedures. First-strand cDNA was synthesized using the Superscript Preamplification System (Gibco BRL) with 0.5–1.0 μg RNA as a template, 2.5 ng $ml^{-1}$ random hexamers, 0.5 mM of each deoxynucleotide triphosphate, 10 mM dithiothreitol and 10 U $\mu l^{-1}$ Superscript RT. The reaction was performed at 42°C for 50 min followed by inactivation of the enzyme at 70°C for 15 min. The samples were treated with 0.1 U $\mu l^{-1}$ *Escherichia coli* RNAase H for 20 min at 37°C, boiled and kept at –20°C. Quantitative PCR analysis of *MDR1* gene expression was performed using oligomers amplifying a 167-bp product; the amounts of template cDNAs were normalized by PCR amplification of $\beta_2$-microglobulin cDNA (internal control) (Noonan and Roninson, 1991). The optimal numbers of PCR cycles were 26 for the *MDR1*-specific product and 20 for the

**Figure 4** PCR analysis and the densitometry of *MDR1* mRNA expression. (**A**) Sublines of mS cells: 1, mS; 2, mS treated with RA (5 μM, 48 h); 3, mS treated with CH (10 ng $ml^{-1}$, 24 h); 4, mS/RAR; 5, mS/RAR treated with RA; 6, mS/RAR treated with CH. (**B**) Sublines of HepG2 cells: 1, Hep/neo; 2, Hep/neo treated with RA; 3, Hep/neo treated with CH; 4, Hep/RAR; 5, Hep/RAR treated with RA; 6, Hep/RAR treated with CH. The 167-bp product band corresponds to *MDR1*; the 120-bp band corresponds to $\beta_2$-microglobulin-specific product (internal control). The density of *MDR1* and $\beta_2$-microglobulin bands were quantified by densitometric scanning on UltroScan Laser Densitometer LKB2202. The results are expressed as the ratio of *MDR1* mRNA to $\beta_2$-microglobulin mRNA. One out of two experiments with similar results is presented

$\beta_2$-microglobulin-specific one. These numbers of cycles yielded clearly detectable PCR products within an exponential range. PCR products were amplified in separate tubes, mixed, resolved by electrophoresis in 7.5% polyacrylamide gel, stained with ethidium bromide, visualized in UV light and analysed by densitometry.

### Northern blot analysis

Total RNA was separated in 1% agarose gel under denaturing conditions according to the method of Lehrach et al (1977). RNA was transferred onto Hybond-N nylon membrane (Amersham,

© Cancer Research Campaign 1998

Buckinghamshire, UK). Nylon membrane was allowed to dry at room temperature and was baked for 1 h at 80°C in a vacuum oven. Blots was hybridized with $^{32}$P-labelled DNA-*RARα* probe as described by Maniatis et al (1982). The plasmid containing a human *RARα* cDNA used for $^{32}$P-labelled probe was provided by Dr SJ Collins (Fred Hutchinson Cancer Research Center). After hybridization, the washed blot was exposed for autoradiography.

### Study of cytotoxicity

The sensitivity of parental cell lines and *RARα* infectants to CH was determined by the colony formation assay. The cells ($2–5 \times 10^2$) were plated onto 60-mm dishes in the medium containing different concentrations of CH and incubated for 14 days. The medium was changed twice a week. The colonies of surviving cells were Giemsa stained. Cell survival was estimated as the ratio of the number of colonies at a given dose of CH to that in control (CH-free) dishes.

### Transmission electron microscopy

The mS cells and their *RARα*-infected counterparts were grown on glass coverslips for 2 days, fixed with 2.5% glutaraldehyde in PBS, post-fixed in 1% osmium tetroxide, dehydrated in a graded ethanol series and embedded into Epon-812. Ultrathin sections were stained sequentially with uranyl acetate and lead citrate and examined in a Geol Elmiscop (GEM-1200exII) at 80 kV.

### The analysis of α-fetoprotein (AFP) expression

Immunochemical detection of AFP in HepG2, McA RH 7777 and their *RARα*-infected variants was performed essentially as described previously (Alekseevskaya et al, 1992). Primary rabbit anti-AFP antibody was a gift from Dr T Eraiser (Cancer Research Centre, Moscow, Russia). Peroxidase-conjugated goat anti-rabbit IgG was used as a secondary antibody. AFP-expressed cells were visualized by enzymatic conversion of diaminobenzidine tetrachloride. The colonies were considered AFP positive (100% stained cells), negative (no staining) or mixed (containing both staining and non-stained variants). Two hundred colonies were counted for each determination.

### RESULTS

### Expression of exogenous *RARα* gene in the infected cells

Full-length cDNA of the human *RARα* was introduced into the mS, HepG2 or McA RH 7777 cells by means of retroviral infection and the sublines mS/RAR, Hep/RAR and 7777/RAR were established. These sublines were isolated as a pool of infected clones in order to avoid clonal variability. The control cells in our experiments were represented by the parental (non-infected) cultures as well as by the sublines with the vector expressing the *neo* gene alone and selected for G418 resistance (*neo* infectants). Transduction of the *neo* gene alone did not change any of the studied characteristics of the cells, e.g. markers of differentiation, constitutive or inducible levels of *MDR1* mRNA or P-gp activity compared with non-infected cells (data not shown). To confirm that the exogenous *RARα* is expressed in G418-resistant cell variants, total RNA from control (*neo* infected) and *RARα*-infected sublines was hybridized with the fragment of human *RARα* cDNA. Figure 1 demonstrates the presence of the exogenous *RARα* transcripts in the sublines of infected cells. These infected cells express the retroviral 4.9 and 3.1 kb *RARα* transcripts (lanes 1, 3 and 4). The mRNA expression of the endogenous *RARα* transcripts in human cells Hep/neo is also visible (lane 2).

**Figure 5** Rh123 efflux by control and *RARα*-infected cells. Flow cytometrical analysis of Rh123 efflux was performed as described in Materials and methods. Logarithmic fluorescence profiles of *RARα*-infected cells (——) and mock-infected (control, – – –). (**A**) mS and mS/RAR cells; (**B**) Hep/neo and Hep/RAR cells; (**C**) 7777/neo and 7777/RAR cells. One representative experiment out of two is shown

### Differentiation and proliferation of RARα-infected cells

The ability to produce melanin is usually considered as a marker of differentiation in melanoma cells (Filippova et al, 1983; Mishima and Imokawa, 1986). To analyse whether the mS and mS/RAR

© Cancer Research Campaign 1998 

**Figure 6** Influence of RA and CH on Rh123 efflux by control and *RARα*-infected cells. Rh123 efflux was analysed after treatment of cells with RA (5 μM, 48 h) (top) and CH (10 ng ml$^{-1}$, 24 h) (bottom). Shown are logarithmic fluorescence profiles of untreated cells (- - -) and treated cells (—). One out of two experiments with similar results is presented cells differ in the production of melanin, we studied these sublines using transmission electron microscopy. The mS/RAR cells became more differentiated as electron microscopy revealed larger quantities of mature premelanosomes in *RARα*-transformed cells than in their parental counterparts (Figure 2A and B). Moreover, RA, a known inducer of melanocyte differentiation, exerted different effects on the parental and *RARα*-infected cells. Treatment of the mS cells with RA (5 μM, 48 h) did not influence

 © Cancer Research Campaign 1998

the differentiation of the cells; in contrast, under the same treatment mS/RAR cells demonstrated an increased number of premelanosomes at all stages of maturation as well as elevation of pigment content (Figure 2C).

We next studied whether the infection of hepatoma cell lines with *RARα* affects the number of cells producing AFP, a marker of hepatocytes in early embryo development. Plating at a low density of the control Hep/neo and 7777/neo resulted in the growth of either AFP-producing (AFP+) or AFP-negative clones (AFP–) as well as the occurrence of the colonies with both AFP+ and AFP– cells (mixed clones). As shown in Figure 3, the number of AFP+ clones significantly decreased in Hep/RAR compared with Hep/neo; also, AFP+ cells almost disappeared from 7777/RAR sublines. These data suggest that the production of AFP is at least partially suppressed in the RARα-infected hepatoma cells.

The RARα-infected human cells proliferated more slowly than the parental cells. The doubling time of mS/RAR and Hep/RAR cells increased in comparison with parental cells (60 h vs 40 h for melanoma cells and 60 h vs 24 h for hepatoma cells), whereas the doubling times of rat 7777/RAR and their parental cells were similar (approximately 24 h). Taken together with the results of ultramicroscopical and immunocytochemical assays, our data indicate that overexpression of exogenous *RARα* rendered the infectants more differentiated.

### *MDR1* gene expression in *RARα*-infected human cells

We next determined the constitutive and inducible levels of *MDR1* mRNA in *RARα*-infected sublines. RT-PCR analysis revealed an increase in the steady-state levels of *MDR1* transcripts in mS/RAR and Hep/RAR compared with mS and Hep/neo respectively (Figures 4A and 4B, compare lanes 1 and 4). Treatment with RA (5 μM, 48 h) resulted in elevation of *MDR1* mRNA levels both in the mS and in the mS/RAR cells (Figure 4A, lanes 2 and 5). The same effect was observed with Hep/neo (Figure 4B, lane 2). The degree of induction was higher in mS/RAR cells, suggesting that in mS/RAR cells the *MDR1* gene became more inducible by RA. In contrast, the levels of *MDR1* mRNA in RA-treated Hep/RAR cells were not higher than in Hep/neo (Figure 4B, lanes 5 and 2). Thus, we did not show the elevation of inducibility of the *MDR1* gene by RA treatment in hepatoma cells.

CH is a P-gp-transported drug shown to induce *MDR1* gene expression in some cell types (Kohno et al, 1989; Chaudhary and Roninson, 1993). Figure 4A (lanes 3 and 6) shows that CH (10 ng $ml^{-1}$, 24 h) did not elevate *MDR1* expression in mS or in mS/RAR cells. However, CH-treated Hep/neo and Hep/RAR cells showed elevation of *MDR1* mRNA expression (Figure 4B, lanes 3 and 6). It is noteworthy that the increase in *MDR1* mRNA in the control Hep/neo treated with CH was significantly greater than in mS treated with the same drugs (Figure 4B, lane 3, compare with Figure 4A, lane 3). This difference may be connected with the tissue origin of the cells: it is known that the level of *MDR1* expression in the liver is comparatively high, whereas the skin is characterized by low amounts of *MDR1* mRNA (Gottesman et al, 1991).

### P-gp activity in the parental and *RARα*-infected cells

P-gp functional activity was measured by means of FACScan analysis of Rh123 efflux from the cells (Neyfakh, 1988). Rh123 is a fluorescent dye that is transported by P-gp out of the cells. The cells with P-gp-mediated MDR exclude Rh123 at a higher rate than drug-sensitive variants; so, the analysis of cell fluorescence after removal of Rh123 from culture medium permits the comparison of P-gp functional activity in different cell populations. The Rh123 technique is very sensitive and allows the detection of initial alterations in P-gp activity (Chaudhary and Roninson, 1992; Egudina et al, 1993).

The results of flow cytometric experiments are presented in Figure 5. Neither of the human *RARα*-infected cell lines studied demonstrated a higher rate of Rh123 efflux than their parental counterparts (Figure 5A and B). In contrast, the comparison of rat hepatoma cells shows that 7777/RAR contained significantly higher amounts of Rh123-dull cells than 7777/neo (Figure 5C). This shows that overexpression of *RARα* did not change P-gp activity in the two studied human cell lines but did change it in the rat cells.

Treatment of *RARα*-infected cells with RA (5 μM, 48 h) led to a dramatic increase in the number of Rh123-dull variants (Figure 6D–F). The effects were demonstrated for all studied cell lines, regardless of their species and tissue origin. However, the induction of P-gp-mediated efflux by RA in mS/RAR cells was less pronounced than that in Hep/RAR or 7777/RAR variants (compare Figure 6D with E and F). Interestingly, treatment of all parental cells with RA did not cause any discernible changes in P-gp function (Figure 6A–C).

CH (10 ng $ml^{-1}$, 24 h) elevated P-gp functional activity in the control cell populations to a greater degree than RA, especially in 7777/neo culture (Figure 6G–I). The effect of CH on P-gp activity was much more pronounced in *RARα* infectants (Figure 6J–L). Again, as with RA treatment, the effect of CH on melanoma cells was lower than in other studied sublines. This elevation of inducibility of *RARα*-infected cells did not necessarily result in the acquisition of CH resistance: the comparison of mS and HepG2 sensitivity with CH revealed an almost identical $IC_{50}$ for parental and infected cells (2–4 ng $ml^{-1}$ CH for both cell types). However, *RARα*-infected rat cells (7777/RAR) became twofold resistant to CH than 7777/neo cells ($IC_{50}$ for 7777/neo cells was 5 ng $ml^{-1}$, and 11 ng $ml^{-1}$ for 7777/RAR cells). These data are in agreement with our results on differences in Rh123 efflux by parental cells and their *RARα*-infected counterparts.

Thus, our data show that the *RARα* gene increases *MDR1* expression but not P-gp activity in transfected cells. However the *RARα* gene elevates inducibility of the function of this protein by the ligand of the RARα (RA) and by the cytotoxic drug (CH).

### DISCUSSION

The goal of our study was to obtain direct evidence of co-ordinated regulation of P-gp-mediated MDR and differentiation in tumour cells and to study some signalling pathways involved in joint regulation of these two cell phenotypes. Previous data show that differentiation and MDR might be connected and that *MDR1*/P-gp expression may be part of the differentiation programme of the cell. However, further studies are needed to prove this supposition (discussed in the Introduction). In this study, we created more differentiated cells by introduction of the gene involved into the cell differentiation programme and investigated the various mechanisms of MDR, i.e. *MDR1* expression, P-gp functional activity and cell resistance to the cytostatic drug.

We isolated the sublines of tumour cells of different species (human and rat) and tissue (hepatoma and melanoma) origin after infection with full-length cDNA of the *RARα* gene. These sublines

© Cancer Research Campaign 1998

were shown to express transgene. As expected, these sublines demonstrated the patterns of more differentiated phenotypes compared with mock-infected counterparts. Elevated amounts of melanin were observed in *RARα*-infected melanocytes; the level of AFP, an embryo-specific liver protein, was decreased in infected hepatocytes. The rate of proliferation of all studied human *RARα*-infectants was slower than that of parental cells. In addition, *RARα*-overexpressing cells appeared to be more sensitive to the induction of differentiation by RA.

The study of *MDR1* gene expression using the highly sensitive RT-PCR technique showed the increased amounts of *MDR1* mRNA in *RARα*-infected human cells. These data suggest that overexpression of *RARα* is the cause of constitutive activation of the *MDR1* gene and/or increase in the stability of *MDR1* mRNA. Our results contrast with previous studies (Teeter et al, 1991) that have shown down-regulation of *MDR1* promoter activity after transient co-transfection of Chinese hamster ovary (CHO) cells with *RARα*- or *RARβ*-expressing vectors together with the *MDR1* promoter region–chloramphenicol acetyltransferase reporter construction. The discrepancy between these and our results could be due to the different cell types used in the experiments. Moreover, the mechanisms of overexpression of an exogenous gene in transiently and stably infected cells may vary (Kopnin et al, 1995; Stromskaya et al, 1995*b*).

We next investigated whether elevated levels of *MDR1* mRNA render *RARα*-infectants more resistant to P-gp-transported compounds. This was studied using two methods: (a) flow cytometric analysis of the efflux of Rh123, a fluorescent dye with high affinity for P-gp, and (b) survival of the cells in the continuous presence of CH, a P-gp substrate. However, neither method revealed any activation of P-gp function in human *RARα*-infectants. These data indicate that overexpression of *RARα* does not lead to the emergence of the MDR phenotype, despite the increase in steady-state levels of *MDR1* mRNA. Several explanations of these data may be proposed. First, the increase in the *MDR1* message is too low to confer discernible levels of MDR. Second, P-gp synthesis may undergo post-transcriptional changes; also, post-translational modifications such as phosphorylation or glycosylation may regulate P-gp-mediated drug transport. In addition, one cannot rule out the possibility that the transport of mature P-gp from the Golgi apparatus might be affected in *RARα* infectants. An alternative possibility is that in *RARα*-infected cells, P-gp has a function unrelated to drug efflux. It is noteworthy that treatment of human neuroblastoma cells with RA induced differentiation and a concomitant overexpression of P-gp, whereas intracellular accumulation of vinblastine, vincristine or actinomycin D was not decreased (Bates et al, 1989). Together with our results, these data suggest that RA-activated signal transduction causes up-regulation of *MDR1* gene expression but does not influence P-gp-mediated drug efflux. If that is the case, P-gp might have other physiological functions in the differentiated cells. It has been postulated that P-gp as well as other ATP-binding cassette transporters can regulate heterologous membrane channels and, perhaps, other membrane proteins (Bates et al, 1989). Probably, in the differentiated cells, P-gp acts as such a regulator or fulfils other functions that are necessary for maintenance of the differentiated phenotype. Recent data suggest that P-gp can function as a lipid flippase of broad specificity that translocates phospholipids across membranes (van Helvoort et al, 1996).

Although the transport of P-gp substrates in non-stimulated *RARα* infectants was unaffected, these sublines were more sensitive to the induction of Rh123 efflux by RA or CH than were parental cells. This activation appeared to be tissue and species specific. In the mS and mS/RAR cells, both CH and RA caused only a slight increase in Rh123 efflux. The continuous exposure of Hep/RAR cells to RA or CH resulted in the significant activation of Rh123 efflux. In the 7777/RAR cells the rate of induction was even higher. Moreover, only these cells among all *RARα* infectants demonstrated occurrence of drug resistance. These data testify that the effects of the overexpression of *RARα* on *MDR1* expression, P-gp transport and MDR are strongly dependent on cell context and are tissue and species specific.

In conclusion, our data provide new evidence that cell differentiation induced by the overexpression of the gene participating in the differentiation programme results in overexpression of the *MDR1* gene and may lead in some cells to elevation of P-gp functional activity and drug resistance. Prolonged treatment of *RARα*-infectants with CH or RA resulted in the increase in both *MDR1* mRNA abundance and Rh123 efflux to a greater extent than in the parental cells. Thus, *RARα* activation increases *MDR1* expression and elevates inducibility of the function of this protein by the cytotoxic drug (CH) and activator (RA) of the transgene. These data imply that differentiation therapy may evoke an important consequence: it may cause the emergence of the MDR phenotype in a portion of the tumour population. Genes involved in cell differentiation and activated in the course of the therapy may elevate the rate of *MDR1*/P-gp response to cytotoxic drugs and thus give these cells selective advantage for survival in the course of chemotherapy.

### ACKNOWLEDGEMENTS

We thank Dr TL Eraizer (Cancer Research Centre, Moscow, Russia) for the generous gift of primary rabbit anti-AFP antibody and Dr EB Mechetner (Irvin, California, USA) for the generous gift of UIC2 MAb. This work was supported by grant 96-04-48485 from the Russian Foundation for Basic Research.

### REFERENCES

Alekseevskaya OD, Anfimova ML, Djuraeva FH, Somova OV, Stavrovskaya AA, Stromskaya TP, Shtil AA and Eraizer TL (1993) Alteration of embryo-specific proteins expression in the multidrug-resistant cells (in Russian). *Herald Cancer Res Center* **2**: 21–30

Bates SE, Mickley LA, Chen YN, Richert N, Rudick J, Biedler JL and Fojo AT (1989) Expression of a drug resistance gene in human neuroblastoma cell lines: modulation by retinoic acid-induced differentiation. *Mol Cell Biol* **9**: 4337–4344

Beck WT and Danks M (1991) Characteristics of multidrug resistance in human tumor cells. In *Molecular and Cellular Biology of Multidrug Resistance in Tumor Cells*, Roninson IB (ed), pp. 3–55. Plenum Press: New York

Becker J, De Nechaud B and Potter VP (1976) Two new rat hepatoma cell lines for studying the unbalanced blocked ontogeny hypothesis. In *Onco-Developmental Gene Expression*, Fishman WH and Sell S (eds), pp 259–270, Academic Press: New York

Biedler JL and Spengler BA (1994) Reverse transformation of multidrug resistant cells. *Cancer Metastas Rev* **13**: 191–207

Chaudhary PM and Roninson IB (1992) Activation of *MDR1* (P-glycoprotein) gene expression in human cells by protein kinase C agonist. *Oncology Res* **4**: 281–290

Chaudhary PM and Roninson IB (1993) Induction of multidrug resistance in human cells by transient exposure to different chemotherapeutic drugs. *J Natl Cancer Inst* **85**: 632–639

Chin K-V, Chauhan SS, Pastan I and Gottesman MM (1990*a*) Regulation of *mdr* RNA levels in response to cytotoxic drugs in rodent cells. *Cell Growth Different* **1**: 361–365

© Cancer Research Campaign 1998

Chin K-V, Tanaka S, Darlington G, Pastan I and Gottesman MM (1990*b*) Heat shock and arsenite increase expression of the multidrug resistance (MDR1) gene in human renal carcinoma cells. *J Biol Chem* **265**: 221–226

Chin K-V, Ueda K, Pastan I and Gottesman MM (1992) Modulation of activity of the promoter of the human *MDR1* gene by *ras* and p53. *Science* **255**: 459–462

Collins SJ, Robertson KA and Mueller LeM (1990) Retinoic acid-induced granulocytic differentiation of HL-60 myeloid leukemia cells is mediated directly through the retinoic acid receptor (RAR-α). *Mol Cell Biol* **10**: 2145–2163

Djuraeva FH, Stavrovskaya AA and Stromskaya TP (1991) Correlations between multidrug resistance and differentiation in the mouse erythroleucosis (in Russian). *Herald Cancer Res Center* **4**: 7–12

Egudina SV, Stromskaya TP, Frolova EA and Stavrovskaya AA (1993) Early steps of P-glycoprotein expression in cell cultures studied with vital fluorochrome. *FEBS Letters* **329**: 63–66

Filippova NA, Parshikova SM and Timar E (1983) Ultrastructure of human skin melanomas (in Russian). *Arch Patologii* **8**: 19–25

Gottesman MM, Willingham MC, Thiebaut F and Pastan I (1991) Expression of the *MDR1* gene in normal human tissues. In *Molecular and Cellular Biology of Multidrug Resistance in Tumor Cells*, Roninson IB (ed), pp. 279–289. Plenum Press, New York

Knowles BB, Howe CC and Aden DP (1980) Human hepatocellular carcinoma cell lines secrete the major plasma proteins and hepatitis B surface antigene. *Science* **209**: 497–499

Kohno K, Sato S, Takano H, Matsuo K-I and Kuwano M (1989) The direct activation of human multidrug resistance gene (*MDR1*) by anticancer agents. *Biochem Biophys Res Commun* **165**: 1415–1421

Kopnin BP, Stromskaya TP, Kondratov RV, Ossovskaya VS, Pugacheva EN, Rybalkina EY, Khokhlova OA and Chumakov PM (1995) Influence of exogenous *ras* and p53 on P-glycoprotein function in immortalized rodent fibroblasts. *Oncol Res* **7**: 299–306

Lehrach J, Diamond D, Wozney J and Boedtker H (1977) RNA molecular weight determinations by gel electrophoresis under denaturing conditions, a critical examination. *Biochemistry* **16**: 4743–4751

Licht T, Ciebig HH, Bross K, Herrmann F, Berger DP, Shoemaker R and Sann M (1991) Induction of multidrug resistance during anti-neoplastic chemotherapy in vitro. *Int J Cancer* **49**: 630–637

Maniatis T, Fritsch EE and Sambrook J (1982) *Molecular Cloning: A Lobaratory Manual*. Cold Spring Harbor Laboratory Press: Cold Spring Herbor, NY

Mickley LA, Bates SE, Richert ND, Currier S, Tanaka S, Foss F, Rosen N and Fojo AT (1989) Modulation of the expression of a multidrug resistance gene (*mdr1*/P-glycoprotein) by differentiating agents. *J Biol Chem* **264**: 18031–18040

Mishima Y and Imokawa G (1986) Melanoma and melanosome genesis. *J Electron Microsc* **35** (suppl. 1): 2153–2156

Neyfakh AA (1988) Use of fluorescent dyes as molecular probes for the study of multidrug resistance. *Exp Cell Res* **174**: 168–178

Noonan K and Roninson IB (1991) Quantitative estimation of *MDR1* mRNA levels by polymerase chain reaction. In *Molecular and Cellular Biology of Multidrug Resistance in Tumor Cells,* Roninson IB (ed), pp. 319–332. Plenum Press: New York

Roninson IB (1991) Structure and evolution of P-glycoprotein. In *Molecular and Cellular Biology of Multidrug Resistance in Tumor Cells*,Roninson IB (ed), pp. 189–211. Plenum Press: New York

Stavrovskaya AA, Stromskaya TP, Chernova OB and Djuraeva FK (1990) Multidrug-resistance and differentiation in the population of transformed canine kidney cells MDCK (in Russian). *Mol Genet Microbiol Virol* **5**: 3–7

Stromskaya TP, Filippova NA, Rybalkina EY, Egudina SV, Shtil AA, Eliseenkova AV and Stavrovskaya AA (1995*a*) Alterations of melanin synthesis in human melanoma cells selected in vitro for multidrug resistance. *Exp Tox Pathol* **47**: 157–166

Stromskaya TP, Grigorian IA, Ossovskaya VS, Rybalkina EY, Chumakov PM and Kopnin BP (1995*b*) Cell-specific effects of RAS oncogene and protein kinase C agonist TPA on P-glycoprotein function. *FEBS Letters* **368**: 373–376

Sugimoto Y and Tsuruo T (1991) Development of multidrug resistance in rodent cells lines. In *Molecular and Cellular Biology of Multidrug Resistance in Tumor Cells*, Roninson IB (ed), pp. 57–70. Plenum Press: New York

Teeter LD, Eckersberg T, Tsai Y and Kuo MT (1991) Analysis of the Chinese hamster P-glycoprotein/multidrug resistance gene pgp1 reveals that the AP-1 site is essential for full promoter activity. *Cell Growth Differ* **2**: 429–437

van Helvoort A, Smiht AJ, Sprong H, Fritzsche I, Schinkel AH, Borst P and van Meer G (1996) MDR1 P-glycoprotein is a lipid translocase of broad specificity, while MDR3 P-glycoprotein specifically translocates phosphatidylcholine. *Cell* **87**: 507–517

© Cancer Research Campaign 1998